# Supplementary material for: Characterisation of Metarhizium majus (Hypocreales: Clavicipitaceae) isolated from the Western Cape Province, South Africa
Source: PLoS One. 2021 Feb 19;16(2):e0240955. doi: 10.1371/journal.pone.0240955 (PMC7894866; doi:10.1371/journal.pone.0240955)
Supplement: Attachment — Submitted filename: Response to Reviewers.docx [file pone.0240955.s001.docx]

**Response to Reviewers**

Additional editors comments:

1-What model has been used for the phylogeny? Why has NJ been used instead of ML? Do they produce the same result? What is the purpose of Bayesian to be used in this study?

- The data were analysed using NJ method using uncorrected “p”. Many fungal studies used NJ for phylogenetic comparisons, as it is more robust and can be combined with Bayesian analysis. The purpose of Bayesian analysis is commonly done to test the probability of nodes forming. The ML tree, Bayesian tree and NJ tree had similar topologies in all cases.

2-In Table 1, if another species sequenced from South Africa needs to be added.

- Page 7: Line 128. An additional *M. majus* isolated, from another locality, have been added to Table 1.

3-Why in Table 1, some locations noted as Brazil and some Brazil, GO? it needs to be clarified. Besides, the outgroup must be added to the table 1.

- Page 7: Line 128. GO next to Brazil deleted. Outgroup added to the table.

4-In the result section, the morphological needs to be improved. The authors must compare with any other morphology or morphometrics of the M. majus reported by others. Information on the morphology and morphometric must be given in a better format and full. Similar species need to be discussed, as well.

- Page 8: Line 132-141.
- Also, the authorship of the species is missed by the authors.
- Page 3: Line 57. Authorship is indicated in the introduction section at the first mention of the species name.

5-In the phylogenetic, the result needs to be rewritten to indicate the clade. The blast result must be provided in which the similarity should be given.

- Page 9: Line 157-167. The different clades were described in the text and the Blastn results provide for identify and coverage.

-I would suggest adding the Genetic Pairwise distance to compare your sequence and the others for the similarity.

- Page 10: Line188-188. Table included on genetic pairwise analysis.

7-In Figure 1 (B and C), the scale is not indicated. By the way, the scale should be the same format for all the figures part.

- Corrected – missing scales indicated and made uniform.

8-The figure's legends need to be improved with such information as a model, frecA, etc.

- Legends improved

9-The quality of Figures 2 and 3 needs to be improved.

- Improved quality.

10-In Phylogenetic trees, the clades must appear on the figures.

- Clades indicated in figures

11-Please check all the citations, figures, and the overall format to be in the PLOS ONE style.

- Formatting checked

Reviewers comments

Reviewer #1:

The authors of the manuscript titled Characterisation of Metarhizium majus (Hypocreales: Clavicipitaceae) isolated from the Western Cape province, South Africa have described the occurrence of an entomopathogenic Metarhizium majus previously unknown to occur in South Africa. The authors carried out pathogenicity assays on two insect pests in addition to morphological and molecular characterization of the fungus.

- Authors should start off by writing in full biological control. This may be shortened later to biocontrol.

Corrected

- Authors should rewrite the abstract. When rewriting this section, authors should take into consideration the relevant parts of an academic abstract.

Page 2: Line 19-30. Abstract adapted

- If If Metarhizium spp. are well-studied entomopathogens, why are the authors not providing examples of other commercialized species of the fungus?

Page 3: Line 44-50. Information added

The authors should also specifically state whether this is a newly identified Metahizium spp., or newly identified as an entomopathogen? Further more, what are the potential benefits of characterizing this new spp. to South Africa and global agriculture?

Page 11: Line 214-215. Information added. Page11: Line 207-209. Stated that it is a new report for SA, not a new species.

- Its interesting that throughout the manuscript authors did not consider stating from which orchards soil samples were collected except in line 127. The authors are encouraged to categorically provide the details of the crop and location of the orchards from where soil samples were collected in Western Cape for isolating Metarhizium spp. Were the soil samples bulked, or analysed separately

Page 4: Line 69-75. Information added

- Line 61: Authors should indicate the common name for Tenebrio molitor

Page 4: Line 78. Information added

- Line 67: Authors should indicate the duration of time for the 75% ethanol and distilled water rinses were carried out

Page 5: Line 83. Information added

- How economically important are these insects to South African agriculture? I expect the authors to have focused on characterizing this entomopathogenic fungus using various economically important insect pests. For example, do these two insect pests cut across all the agricultural production regions of South Africa? Are they important constraints to the production of food staples? Do they vector important diseases? These pests are in short not representative enough.

Focus are on characterisation and occurrence of *M. majus* not the insects.

- Line 81: Authors should write in full.

Page 5: Line 97. Corrected

- Line 92: Percent concentration of agarose gel? Voltage used and duration of time for which it was run. What imaging system was used to document the results of the electrophoresis?

Page 6: Line 109-110. Corrected

- Line 95: BLASTn analysis was carried out on...

Page 6: Line 112. Corrected

- Lines 96-97: Fungal cultures were deposited in the fungal collection of the Biosystems Division of the Agricultural Research Council (ARC), Pretoria.

Page 6: Line 113-115. Corrected

- What about the partial sequences of the tubulin (BtuB)

The sequences of Btub were included in the dataset of fig 2. Few sequences available.

- Line 115: What taxonomic key was used for morphological identification?

Page 8: Line 132-142.Information added, however, we relied mainly on molecular identification of the EPF isolate, as the *M. anisopliae* species complex species mostly look similar morphologically.

- Lines 152-153: Authors should provide a citation

Page 10: Line 193. Corrected

- Line 166: Authors should italicize M. majus

Page 11: Line 206. Corrected

**Journal Requirements**:

In your Methods section, please provide additional information regarding the permits you obtained for the work. Please ensure you have included the full name of the authority that approved the field site access and, if no permits were required, a brief statement explaining why.

Page : Line 71-73. Information added
