# Supplementary material for: Characterisation of Metarhizium majus (Hypocreales: Clavicipitaceae) isolated from the Western Cape Province, South Africa
Source: PLoS One. 2021 Feb 19;16(2):e0240955. doi: 10.1371/journal.pone.0240955 (PMC7894866; doi:10.1371/journal.pone.0240955)
Supplement: Attachment — Submitted filename: Response to Reviewers.docx [file pone.0240955.s002.docx]

**Response to Reviewers**

Reviewer #1:

Lines 26-27: M. majus and T. molitor should be written in full the first time they are being introduced.

-	Page 2: Line28-30. Corrected

Lines 44-46: Similar to orthoptera, hemiptera and thysanoptera, cite examples for lepidoptera, coleoptera, diptera and hymenoptera.

-	Page 3: Line 47-49. Corrected

Line 69: ...from the surveyed plum, apricot, and quince orchards in Tierhoek farm. Soils were obtained at a depth of 15 cm from under the tree canopy.

-	Page 4: Line 72-73. Changed as suggested

Line 73: It may not be relevant mentioning the name of the orchard owner at this juncture.

-	Name of the orchard owner removed.

Lines 76-77: wax moth repeated twice

-	Corrected

Lines 93-100: what is the taxonomic reference (identification key) that was used for the morphological identification? Although the authors have indicated their reliance on molecular identification, morphological identification is an important aspect of taxonomic classification. If it is an experiment carried out in this study, it carries similar importance as other experiments.

-	Page 5; Line 105-106. Morphological of identification of the entomopathogenic fungi was done by according to the key Humber 2012. Add to reference list: 14.	Humber RA. Identification of entomopathogenic fungi. In: Lacey LA, editor. Manual of techniques in invertebrate pathology. 2nd ed. Burlington: Elsevier; 2012. pp. 151-187. https://doi.org/10.1016/B978-0-12-386899-2.00006-3

Line 104: The polymerase chain reaction (PCR) was conducted...

-	Page 5: Line 110. Corrected

Line 110: From what template were the sequences generated? It appears a critical step in the methodology has been ommitted.

-	Page 5-6: Line 110-112. A polymerase chain reaction (PCR) was conducted, using the KAPA2G Robust HotStart ReadyMix [KAPA Taq EXtra HotStart DNA Polymerase, KAPA Taq EXtra Buffer, dNTPs (0.3 mM of each dNTP), MgCl_2_ (2 mM at 1X) and stabilisers] PCR kit.

Line 118: ...combining 'concatenate' may be a better word to use. In addition, the same synonyms used in the methods section should be used for ITS, BtuB, and EF-1α genes.

-	Page 6: Line 127. Corrected as suggested

Lines 127-128: the same headers listed in line 128 should be used as headers for table 1. In addition include footnotes to table 1 describing in full ITS, BtuB, and EF-1α

-	Page 7: Line 138-139. Corrected throughout text

Line 166: 'The South African M. majus did not differ in base similarity from the type strain (KJ398801) of the EF-1α gene, and differs by ...% (indicate percentage) from the most closely related M. guizhouense.

-	Page 9: Line178-181. Corrected as suggested

Lines 185-189: include footnotes in table 2. For example what does 1 to 14 represent?

-	The cell-column with the numbers have been merged with those of the species name to indicate that the number correspond with the horizontal heading of the matrix, which show the standard error in the above diagonal.

The manuscript should be thoroughly checked for grammatical errors. The style of writing and formatting used in other relevant manuscripts an be followed. Articles published in this reputable journal will be suitable.

-	Language edited by a professional language editor, style thoroughly checked and the discussion improved
