# Supplementary material for: Characterisation of Metarhizium majus (Hypocreales: Clavicipitaceae) isolated from the Western Cape Province, South Africa
Source: PLoS One. 2021 Feb 19;16(2):e0240955. doi: 10.1371/journal.pone.0240955 (PMC7894866; doi:10.1371/journal.pone.0240955)
Supplement: Attachment — Submitted filename: Response to Reviewers.docx [file pone.0240955.s003.docx]

Response to Reviewer’s

The referee commented on the Discussion to be improved based on your finding. Revisions 2 and 3 submitted previously have not significant differences. Therefore, I will suggest you improve the Discussion for finalizing your manuscript.

- Improvements have been made to the discussion
