# Supplementary material for: Characterisation of Metarhizium majus (Hypocreales: Clavicipitaceae) isolated from the Western Cape Province, South Africa
Source: PLoS One. 2021 Feb 19;16(2):e0240955. doi: 10.1371/journal.pone.0240955 (PMC7894866; doi:10.1371/journal.pone.0240955)
Supplement: Attachment — Submitted filename: Response to Reviewers.docx [file pone.0240955.s005.docx]

**Response to Editor’s comments**

Thank you for including your ethics statement on the online submission form: "Soil samples were collected from the orchards surveyed plum, apricot and quince, at a depth of 15 cm, from under the tree canopy on Tierhoek farm (-33˚13'19.687''S 19˚38'44.281'') (- 33,711596; 19,790730), in Tierhoek Valley near Robertson in the Western Cape province. A permit for collection of soil samples at the farm was issued by the farm owner and manager B.K.C. Gilson." To help ensure that the wording of your manuscript is suitable for publication, would you please also add this statement at the beginning of the Methods section of your manuscript file.

- Statement added and rephrased

The files all received. The main text is acceptable but I have corrected it with minor changes especially your affiliation which was not in the main text.

- Minor changes have been accepted in the text and the affiliation corrected

The GPS coordinates are not correct and not showing the location mentioned in the manuscript.

- GPS reading corrected

All changes are in yellow and please check the yellow highlight and then send it back to me as soon as possible to finalize the decision.

- All changes in yellow checked

The phylogenies figures from my side are getting blur and I have maximized the dpi to 500. If you have better quality please send them to me. The LM picture is acceptable.

- The two phylogenetic trees have being redone to improve the quality

From the editor:

I have checked up the manuscript submitted by you and your colleagues. I am pleased to inform you that your manuscript was accepted for publication in PLOS ONE.
